# Depression or anxiety disorder moderates the relationship between smoking status and e-cigarette use status: a cross-sectional study

**DOI:** 10.1186/s12889-025-24983-4

**Published:** 2025-10-29

**Authors:** Tareq F. Alotaibi, Mohammed M. Alqahtani

**Affiliations:** 1 Department of Respiratory Therapy, College of Applied Medical Sciences, King Saud bin Abdulaziz University for Health Sciences, Riyadh, Saudi Arabia; 2 King Abdullah International Medical Research Center, Riyadh, Saudi Arabia; 3 Department of Respiratory Services, King Abdulaziz Medical City, Ministry of National Guard Health Affairs, Riyadh, Saudi Arabia

**Keywords:** Smoking status, Electronic cigarette, Depression, Anxiety disorder, Adults

## Abstract

**Background:**

Electronic cigarettes (e-cigarettes) deliver nicotine by vaporizing nicotine-containing fluids without involving tobacco combustion and are available in various flavors. In 2021, the age-standardized prevalence of current e-cigarette use among U.S. adults was 6.9%, with nearly half of these individuals using e-cigarettes daily (Erhabor et al, JAMA Netw Open.6:e2340859, 2023). While there is a well-documented relationship between depressive and anxiety disorders and combustible cigarette smoking, less is known about how these mental health conditions relate to e-cigarette use. This study aimed to examine the role of depression or anxiety disorders as moderators of the relationship between smoking status and e-cigarette use among adults.

**Methods:**

This cross-sectional study used data from the Health Information National Trend Survey (HINTS), Cycle 2, 2018, for secondary data analysis. The HINTS data are nationally representative of adults of adults living in the United States. The independent variable in this study was smoking status (current, former, or never smoked). The dependent variable was e-cigarette use status (current, former, or never). We used SPSS and the PROCESS macro for multinomial logistic regression, assessing e-cigarette use impacts from smoking status and mental health, with significance at *p* ≤ 0.05.

**Results:**

The logistic regression showed that smoking status, depression or anxiety, and their interaction predicted e-cigarette use better than chance (F_(6, 2980)_ = 120.1, *p* < 0.001, *R*^*2*^ = 0.195). Smoking status was associated with increased odds of e-cigarette use (OR, 1.44; 95% CI 1.34–1.55). Depression or anxiety was also associated with increased odds of e-cigarette use (OR, 1.31; 95% CI 1.18–1.44). The interaction between smoking status and depression or anxiety was significant (*b* = -0.081, *t*_(2980)_ = -3.85, *p* = 0.0001).

**Conclusion:**

This study showed that depression or anxiety disorders moderated the relationship between smoking status and e-cigarette use. These findings may inform the development of targeted, theory-based interventions aimed at reducing e-cigarette use among individuals with mental health conditions.

## Background

Tobacco smoking is recognized as one of the most critical global health challenges. Between 1960 and 2020, it was responsible for approximately 41 million deaths in the United Kingdom and North America [1]. According to the World Health Organization, tobacco use is estimated to cause more than 8 million deaths annually, including around 1.3 million non-smokers who die due to exposure to second-hand smoke [2]. Tobacco smoking is a major risk factor for the development of several respiratory illnesses, including asthma, chronic obstructive pulmonary disease (COPD), tuberculosis, and lung cancer [3].

Electronic cigarettes (e-cigarettes) deliver nicotine by vaporizing nicotine-containing fluids, without involving tobacco combustion, and are available in a variety of flavors [4]. They have emerged as novel nicotine delivery devices that provide users with an experience similar to conventional tobacco smoking [4]. The tobacco industry has introduced and aggressively marketed these products to sustain and promote nicotine addiction. As a result, greater attention should be directed toward eliminating all forms of tobacco use through comprehensive tobacco control strategies [2]. These strategies include public media campaigns, smoke-free policies, increased taxation, enhanced access to tobacco cessation programs, and stricter regulations on the sale and marketing of tobacco products [2].

Various types of e-cigarettes have been developed, differing in the solutions used to generate nicotine aerosols and in their aerosol volumes. These devices typically use carrier mixtures such as propylene glycol, with or without glycerol [4]. E-cigarettes are available in a wide range of flavors, and users can purchase the devices, e-liquids, or e-juices separately, with varying concentrations of nicotine [4]. However, growing evidence indicates that e-cigarettes pose significant health risks due to the presence of harmful substances, including carcinogenic metabolites and toxic compounds such as formaldehyde, acetaldehyde, acrolein, propanol, acetone, and butanal. Studies have shown that the levels of these chemicals are higher in e-cigarette users compared to traditional cigarette users [5, 6]. Notably, formaldehyde and acetaldehyde are classified as probable human carcinogens, while acrolein is a known respiratory irritant that can induce lung inflammation and contribute to the development of chronic obstructive pulmonary disease (COPD) [7, 8].

Despite the known health risks of e-cigarettes, their prevalence continues to rise among adults. One study reported the prevalence of current e-cigarette use among adults, stratified by age groups, as follows: 18–20 years (15.3%), 21–24 years (19.2%), 25–29 years (15.9%), 30–34 years (13.6%), 35–39 years (8.4%), 40–44 years (7.9%), 45–49 years (4.4%), 50–54 years (4.6%), 55–59 years (3.8%), and ≥ 60 years (7.0%) [9]. The use of e-cigarettes as a tool for smoking cessation remains a topic of ongoing debate. For example, a systematic review and meta-analysis found that the use of nicotine-containing e-cigarettes was associated with a significant increase in smoking abstinence, as measured by the most rigorous definition of abstinence reported (risk ratio [RR] = 1.77; 95% confidence interval [CI]: 1.29–2.44) [10]; however, they are also perceived as a potential gateway to the use of conventional cigarettes, cannabis, and opioids [11]. Additionally, research indicates that relapse rates among e-cigarette users attempting to quit smoking are high, with many transitioning to dual use of e-cigarettes and combustible cigarettes [12]. This pattern of dual use can undermine the potential benefits of smoking cessation and perpetuate nicotine addiction, underscoring the challenges of long-term quitting and the need for more comprehensive intervention strategies [12].

E-cigarette use is considered a complex behavior due to the interplay of biological, psychological, social, and environmental factors [13]. It is important to coordinate efforts to examine the extent to which e-cigarettes are used by individuals who have never smoked, as well as by current and former smokers. The perception that e-cigarettes pose a lower health risk than conventional cigarettes may contribute to increased use and the associated harmful consequences of nicotine addiction and smoking [14–16].

Tobacco smoking is globally recognized as a major risk factor for numerous health conditions and frequently co-occurs with mental health disorders such as depression and anxiety. These comorbidities can exacerbate smokers’ risk profiles and influence their patterns of e-cigarette use [16, 17]. Research indicates that mental health conditions are associated with earlier initiation, heavier consumption, and greater nicotine dependence [18]. Specifically, conditions like generalized anxiety disorder (GAD) and major depressive disorder (MDD) are linked to higher smoking prevalence and lower cessation success rates. While the relationship between depressive and anxiety disorders and traditional cigarette smoking is well documented, the mechanisms underlying e-cigarette use among individuals with these conditions remain poorly understood, revealing a significant gap in the literature. This highlights the need for targeted research examining how depression and anxiety influence smoking behaviors and e-cigarette use across diverse populations [19–22]. Notably, few studies have explored whether specific mental health conditions, such as depression or anxiety, moderate the relationship between traditional smoking and e-cigarette use. This lack of evidence presents a critical gap in understanding the interplay between mental health and nicotine-related behaviors—knowledge that is essential for designing effective, tailored interventions.

Increasing attention has been directed toward understanding how psychological disorders, such as depression, influence smoking initiation and sustained tobacco use. Research suggests that depressive symptoms are strong predictors of smoking persistence among adults [20]. However, there remains a significant gap in knowledge regarding how these disorders affect susceptibility to e-cigarette use across different smoking statuses—from current and former smokers to those who have never smoked.

**Fig. 1:**
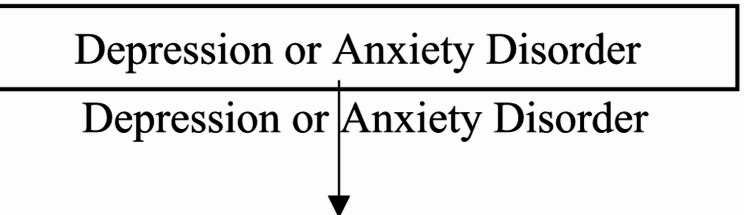
Conceptual framework for the relationship between the smoking status and electronic cigarette use, and how depression or anxiety disorder moderate this relationship

This study aims to address these gaps by examining the moderating effects of depression and anxiety on the relationship between smoking status and e-cigarette use. The findings are intended to inform effective public health interventions aimed at reducing both traditional and electronic cigarette use by analyzing how psychological factors influence e-cigarette initiation and demand. Our goal is to clarify the interactions between mental health conditions and smoking behaviors, thereby contributing to the development of targeted tobacco control strategies and prevention efforts. We hypothesize that the presence of a depressive or anxiety disorder moderates the relationship between smoking status and e-cigarette use status, after controlling for demographic variables such as age, gender, and education (See Fig. 1).

## Methods

### Study design

This study was conducted using a cross-sectional design, analyzing self-reported data from the Health Information National Trends Survey (HINTS). HINTS is a nationally representative survey that collects information on the American public’s use and access to health information [23, 24].

### Participants

This cross-sectional study utilized data from the Health Information National Trends Survey (HINTS), Cycle 2 (2018), for secondary data analysis. HINTS 2018 is the most recent version of the survey and is nationally representative of the U.S. adult population [23]. The inclusion criteria for HINTS ensure a representative sample of U.S. adults. Participants must be 18 years or older, reside in non-institutionalized settings within the United States (e.g., private residences), and be able to read and understand either English or Spanish, as the survey is administered in both languages. The sampling process employs a randomized, address-based method, with one adult randomly selected per household to participate voluntarily [23]. Exclusion criteria include individuals under the age of 18, residents of institutionalized settings (e.g., nursing homes or correctional facilities), and those who cannot comprehend English or Spanish. These criteria are designed to ensure a diverse and inclusive cross-section of the U.S. population for examining health communication trends and disparities [23].

### Sampling procedure

HINTS 5, Cycle 2, utilized a two-stage sampling design. First, a stratified sample of residential addresses was selected from all residential areas. These addresses were provided by the Marketing Systems Group (MSG). The sampling frame was stratified into two categories: areas with a high minority concentration and areas with a low minority concentration. High-minority areas were defined as census tracts where the combined proportion of residents identifying as Hispanic or African American was 34% or greater, while low-minority areas included all other tracts with less than 34% Hispanic or African American population [23].

An equal probability sample of addresses was selected from each stratum to ensure adequate representation. In the second stage, one adult respondent was selected from each sampled household using the Next Birthday Method—a commonly used approach in population surveys that selects the adult (aged 18 or older) in the household whose birthday is next on the calendar. This method helps randomize selection within households and minimizes selection bias. Each selected adult was invited to participate in the survey via postal mail and was offered a $2 cash incentive to encourage participation [23]. Bilingual support was provided through two toll-free telephone numbers: one for English speakers and one for Spanish speakers. A total of 14,586 households received mailed invitations to participate in the survey. The overall household response rate was 32.9% [23].

After participants returned their surveys, all questionnaires were reviewed to determine eligibility for inclusion in the final dataset, as outlined in the HINTS 5, Cycle 2 methodology report [23]. A total of 3,547 questionnaires were received, of which 3,504 were deemed eligible. Among the 43 ineligible responses, two were submitted by individuals under the age of 18, and two were excluded due to suspicious response patterns suggesting the respondents were not truthful. According to the methodology report, these responses showed evidence of fabrication or invalid response behavior, such as inconsistent or implausible answers throughout the survey. Additionally, 19 surveys were incomplete, and 20 were identified as duplicates from the same household. These screening procedures are part of HINTS’ standard data quality assurance protocol [23]. For the purposes of this secondary analysis, we included only participants with complete data on all key study variables, including smoking status, e-cigarette use, and depression or anxiety status. This resulted in a final analytic sample of 3,255 participants. The difference between the 3,504 eligible participants and the final analytic sample is due to listwise deletion by the statistical software, which automatically excludes cases with missing responses on any of the variables used in the regression models. These missing responses primarily affected outcome or moderator variables and were not uncommon given the sensitive nature of the questions. Further details regarding the sampling design, data collection procedures, and quality assurance protocols can be found in the HINTS 5 Cycle 2 Methodology Report, which outlines the rigorous approach used to ensure the representativeness and accuracy of the survey data [available at: https://hints.cancer.gov/].

### Ethics

All individuals who participated in the original HINTS study provided informed consent. The HINTS 5 protocol was approved by the Westat Institutional Review Board. Additionally, the study was granted an exemption from further IRB review by the NIH Office of Human Subjects Research Protections. As this study utilized publicly available, de-identified data from HINTS, it was exempt from requiring informed consent or additional ethical approvals for secondary analysis [23]. Furthermore, we obtained exempt (expedited) IRB approval (NRR24/108/12) from King Saud bin Abdulaziz University for Health Sciences, Riyadh, Saudi Arabia, to permit the use of these data.

### Measures

#### E-cigarette use status

E-cigarette use status was evaluated as the dependent variable. It was assessed through a question asking whether the participant had ever used an e-cigarette, even once or twice, with response options of “Yes” or “No.” Participants who responded “Yes” were then asked a follow-up question about how often they currently used e-cigarettes, with the following response options: “Every day,” “Some days,” or “Not at all.”

Based on responses to these two questions, participants were classified into three categories:

- Non-users: Responded “No” to the first question.
- Current e-cigarette users: Responded “Yes” to the first question and either “Every day” or “Some days” to the second.
- Former e-cigarette users: Responded “Yes” to the first question and “Not at all” to the second.

#### Smoking status

Smoking status was treated as the independent variable. It was assessed through a question asking whether the participant had smoked at least 100 cigarettes in their lifetime, with response options of “Yes” or “No.” Participants who answered “Yes” were then asked a follow-up question about their current smoking frequency, with the options: “Every day,” “Some days,” or “Not at all.”

Based on responses to these two questions, participants were categorized into three groups:

- Never smokers: Responded “No” to the first question.
- Current smokers: Responded “Yes” to the first question and “Every day” or “Some days” to the second question.
- Former smokers: Responded “Yes” to the first question and “Not at all” to the second question.

#### Covariates

Participants’ age, sex, and education level were collected as demographic variables. Gender was treated as a categorical variable (Male, Female); age was measured as a continuous variable; and education level was treated as a categorical variable with the following categories: less than 8 years, 8–11 years, 12 years or completed high school, post-high school training other than college (e.g., vocational or technical), some college, college graduate, and postgraduate.

#### Moderators

Depression or anxiety disorder was assessed by asking participants whether a doctor or other health professional had ever told them that they had been diagnosed with depression or an anxiety disorder (single variable.) Responses were a single variable and categorized as “Yes” or “No.

### Data analysis

SPSS version 24 was used for data analysis. Descriptive statistics, including means, standard deviations, ranges, and frequency distributions, were computed. Regression analyses were conducted using the PROCESS macro developed by Hayes. A *p*-value of < 0.05 was considered statistically significant. The PROCESS macro includes pre-programmed models, each identified by a number corresponding to a specific analytical approach; for instance, Model 1 is used for moderation analysis, while Model 4 is used for mediation analysis [25]. When using the PROCESS macro, there was no need to dummy code multicategorical independent variables into k–1 variables, as the macro accepts them in their original form. By default, the macro treats the group with the lowest frequency as the reference group for multicategorical variables [25]. The variables were retained as originally coded for this study. Smoking status was categorized as current smokers (1), former smokers (2), and never smoked (3), while depression or anxiety status was coded as yes (1) and no (2). Accordingly, current smokers and participants with depression or anxiety were treated as the reference groups, as they represented the categories with the fewest participants. To account for the multicategorical nature of the outcome variable—e-cigarette use status (current user, former user, never user)—a multinomial logistic regression model was employed. The analysis utilized Hayes’ PROCESS macro, which enables the modeling of interaction effects within a multinomial framework. In this model, “never used e-cigarettes” served as the reference group. The model examined the effects of smoking status and depression/anxiety status on the likelihood of being a current or former e-cigarette user. Centering was not applied to the independent variable (smoking status) or the moderator (depression/anxiety status), as both were categorical variables.

## Results

Descriptive statistics for all variables are presented in Table 1. A total of 3,255 participants with complete data on all relevant variables were included in the final analysis. The sample comprised more women than men (58.3% vs. 39.7%, respectively). Participants ranged in age from 18 to 101 years (M = 56.34, SD = 16.14), with the majority aged 66 years or older. Approximately 25% of the sample were college graduates, while only 1.7% had less than 8 years of education. Over 75% of participants reported not having anxiety or depression, and about 60% had never smoked. Additionally, 82% of the sample had never used e-cigarettes, while 2.4% were current e-cigarette users.

**Table 1:** Sociodemographic characteristics of the Participants*

	Valid *N*	Percentage	Minimum	Maximum
Gender			1	2
Men =1	1303	39.7%		
Women =2	1941	58.3%		
Age (years)	3146		1	6
≤ 25 = 1	97	3%		
26–35 = 2	314	9.6%		
36–45 = 3	412	12.5%		
46–55 = 4	556	16.9%		
56–65 = 5	802	24.4%		
≥ 66 = 6	1104	33.6%		
Education			1	7
Less than 8 years =1	56	1.7%		
8–11 years =2	161	4.9%		
12 years or high school = 3	616	18.8%		
Post high school other than collage = 4	228	6.0%		
Some collage = 5	714	21.7%		
College graduate = 6	828	25.2%		
Postgraduate = 7	578	17.6%		
Depression/anxiety			1	2
Yes = 1	730	22.2%		
No = 2	2484	75.6%		
Smoking status			1	3
Current = 1	414	12.6%		
Former = 2	856	26.1%		
Never = 3	1985	60.4%		
e-cigarette use			1	3
Current = 1	80	2.4%		
Former = 2	282	8.6%		
Never = 3	2831	86.2%		

*The total sample size (*N* = 3,255) reflects participants with complete data on key study variables, including demographics, smoking status, e-cigarette use, and mental health indicators. Due to item-level nonresponse, the number of respondents may vary slightly across individual variables

Logistic regression analysis indicated that smoking status, depression or anxiety, and their interaction significantly predicted e-cigarette use above chance levels, F(6, 2980) = 120.1, *p* < 0.001, R² = 0.195. As shown in Table 2, age was significantly positively associated with e-cigarette use (*b* = 0.004, SE = 0.0004, *t*(2980) = 9.85, *p* < 0.001). Smoking status was significantly associated with increased odds of e-cigarette use (OR = 1.44; 95% CI: 1.34–1.55), as was the presence of depression or anxiety (OR = 1.31; 95% CI: 1.18–1.44).

**Table 2:** Influence of anxiety or depression on the relationship between e-cigarette use and smoking status. **

			Predictor Variables		
	Intercept	*b*	*SE*	*t*	*P*
	2.65				
Gender		−0.009	0.014	−0.65	0.52
Age		0.004	0.0004	9.85	< 0.001
Education		−0.0018	0.004	−0.41	0.68
Smoking status		0.22	0.009	9.81	< 0.001
Depression/anxiety		0.065	0.017	3.9	0.0001
Smoking * anxiety		−0.081	0.021	−3.85	0.0001

**Analyses were conducted using listwise deletion. The final analytic sample includes only participants with complete data on all variables included in the model. Cases with missing data were automatically excluded

The interaction between smoking status and depression or anxiety was statistically significant (*b* = −0.081, SE = 0.02, *t* (2980) = −3.85, *p* = 0.0001; see Table 2), suggesting that depression or anxiety may play a moderating role in the relationship between smoking and e-cigarette use. The conditional effects analysis from the PROCESS macro indicated that smoking status significantly predicted e-cigarette use both in the presence (*b* = 0.28, *t* = 16.01, *p* < 0.001) and absence (*b* = 0.20, *t* = 17.3, *p* < 0.001) of depression or anxiety. These findings suggest that the association between smoking and e-cigarette use is stronger among individuals with depression or anxiety.

## Discussion

Few studies have assessed the prevalence of e-cigarette use across smoking status categories, including current smokers, former smokers, and individuals who have never smoked. Even fewer have explored the moderating role of depression or anxiety disorders in the relationship between smoking status and e-cigarette use. The findings of this study are consistent with prior research indicating that e-cigarette use is prevalent across all smoking status groups (current, former, and never smokers) [9, 26, 27]. This study found that individuals who had never smoked and former smokers reported higher rates of e-cigarette use compared to current cigarette smokers. This finding aligns with the results of Dutra and Glantz [28], who reported that e-cigarettes were more appealing to never smokers and former smokers. Additionally, age emerged as the only demographic variable that significantly predicted e-cigarette use, consistent with previous research indicating that e-cigarette use is more prevalent among young adults [9, 26, 27]. Furthermore, this study demonstrated that having a depression or anxiety disorder was a significant predictor of e-cigarette use, corroborating earlier findings that e-cigarette use is more common among individuals with poor mental health compared to those without depression or anxiety [26, 29, 30], which showed that e-cigarette use was more prevalent among individuals with poor mental health compared to those without depression or anxiety.

Pre-existing trait markers—such as personality traits, trait anxiety, and depression—are important in explaining inter-individual differences and are useful for identifying the underlying sources of variation at the population level [31]. In this study, the interaction between depression or anxiety disorder and smoking status was statistically significant, indicating that the relationship between smoking status and e-cigarette use was moderated by the presence of depression or anxiety. Specifically, the interaction analysis revealed that current smokers were more likely to use e-cigarettes, particularly those without depression or anxiety. In contrast, former smokers exhibited a greater likelihood of e-cigarette use when they had depression or anxiety, compared to those without such conditions. Interestingly, among never smokers, e-cigarette use was highest overall, with no significant difference observed between those with and without depression or anxiety. These nuanced findings underscore the need for further research to deepen our understanding of the complex interplay between mental health status, smoking behavior, and e-cigarette use.

This study has several methodological limitations. First, its cross-sectional design does not allow for causal inference. Therefore, the findings should be interpreted with caution and ideally replicated using longitudinal data with repeated measures of mental health status. Such designs could offer greater insight into temporal and causal relationships while minimizing recall and reporting biases. Additionally, further research with larger and more diverse samples is needed to enhance the reproducibility and generalizability of the results.

Second, this study was limited to data from the United States, and it remains unclear whether similar patterns would be observed in other countries. Future research should explore whether these findings hold true in different cultural and geographic contexts. Third, the study did not account for the influence of other important sociodemographic variables, such as race, ethnicity, and income. Future investigations should consider these factors to better understand their interactions with smoking status, mental health conditions, and e-cigarette use.

Another key limitation is that depression and anxiety disorders were not assessed using validated psychometric instruments, DSM criteria, or medically confirmed diagnoses. Instead, mental health status was based on a self-reported item, which may be prone to misclassification. This limitation is particularly concerning, given that many individuals—including health professionals—may lack the qualifications to accurately diagnose psychological disorders. To improve validity, future research should incorporate standardized diagnostic tools or clinical assessments. Finally, biochemical verification of tobacco or nicotine use was not conducted, which may limit the accuracy of self-reported smoking behaviors. Despite these limitations, this study contributes to the growing body of literature on the psychological determinants of smoking behavior and e-cigarette use.

## Conclusions

In summary, this study found that the interaction between smoking status and depression or anxiety disorders significantly predicted e-cigarette use. As e-cigarette use continues to rise, these findings can inform the development of theory-based interventions that address the complex relationship between mental health and tobacco use behaviors. The study offers valuable insights for researchers, public health professionals, healthcare providers, and regulatory authorities aiming to understand current trends in e-cigarette use among current smokers, former smokers, and never smokers.

## Data Availability

The data used in this study are publicly available and can be accessed from the HINTS database at https://hints.cancer.gov/.

## Abbreviations

HINTS: Health Information National Trend Survey

## References

[1] Jha P. The hazards of smoking and the benefits of cessation: a critical summation of the epidemiological evidence in high-income countries. Elife. 2020. 10.7554/eLife.49979. 32207405 10.7554/eLife.49979 PMC7093109

[2] Organization WH. Tobacco. World health Organization; n.d. Fact Sheet. Cited 2024.

[3] Gan H, Hou X, Zhu Z, Xue M, Zhang T, Huang Z, et al. Smoking: a leading factor for the death of chronic respiratory diseases derived from global burden of disease study 2019. BMC Pulm Med. 2022;22(1):149. 35443660 10.1186/s12890-022-01944-w PMC9019969

[4] Kathuria H. Electronic cigarette use, misuse, and harm. Med Clin North Am. 2022;106(6):1081–92. 36280334 10.1016/j.mcna.2022.07.009

[5] Shahab L, Goniewicz ML, Blount BC, Brown J, McNeill A, Alwis KU, et al. Nicotine, carcinogen, and toxin exposure in long-term E-cigarette and nicotine replacement therapy users: a cross-sectional study. Ann Intern Med. 2017;166(6):390–400. 28166548 10.7326/M16-1107 PMC5362067

[6] Taylor E, Simonavičius E, McNeill A, Brose LS, East K, Marczylo T, et al. Exposure to tobacco-specific nitrosamines among people who vape, smoke, or do neither: a systematic review and meta-analysis. Nicotine Tob Res. 2024;26(3):257–69. 37619211 10.1093/ntr/ntad156 PMC10882431

[7] Institute NC. Formaldehyde and Cancer Risk 2024 Available from: https://www.cancer.gov/about-cancer/causes-prevention/risk/substances/formaldehyde/formaldehyde-fact-sheet.

[8] Sheth P, Mehta F, Jangid G, Anamika FNU, Singh B, Kanagala SG, Jain R. The rising use of E-Cigarettes: unveiling the health risks and controversies. Cardiol Rev. 2024. 10.1097/CRD.0000000000000666. Epub ahead of print. PMID: 38385663. 10.1097/CRD.0000000000000666 38385663

[9] Erhabor J, Boakye E, Obisesan O, Osei AD, Tasdighi E, Mirbolouk H, et al. E-cigarette use among US adults in the 2021 behavioral risk factor surveillance system survey. JAMA Netw Open. 2023;6(11):e2340859. 37921768 10.1001/jamanetworkopen.2023.40859 PMC10625038

[10] Levett JY, Filion KB, Reynier P, Prell C, Eisenberg MJ. Efficacy and safety of E-Cigarette use for smoking cessation: A systematic review and Meta-Analysis of randomized controlled trials. Am J Med. 2023;136(8):804–e134. 37148992 10.1016/j.amjmed.2023.04.014

[11] Chen G, Rahman S, Lutfy K. E-cigarettes may serve as a gateway to conventional cigarettes and other addictive drugs. Adv Drug Alcohol Res. 2023;3:11345. 38389821 10.3389/adar.2023.11345 PMC10880776

[12] Hartmann-Boyce J, McRobbie H, Lindson N, Bullen C, Begh R, Theodoulou A, et al. Electronic cigarettes for smoking cessation. Cochrane Database Syst Rev. 2021;4(4):Cd010216. 33913154 10.1002/14651858.CD010216.pub5 PMC8092424

[13] Glasser AM, Collins L, Pearson JL, Abudayyeh H, Niaura RS, Abrams DB, et al. Overview of electronic nicotine delivery systems: A systematic review. Am J Prev Med. 2017;52(2):e33–66. 27914771 10.1016/j.amepre.2016.10.036 PMC5253272

[14] Gülşen A, Uslu B. Health hazards and complications associated with electronic cigarettes: a review. Turk Thorac J. 2020;21(3):201–8. 32584238 10.5152/TurkThoracJ.2019.180203 PMC7311157

[15] National Academies of Sciences E, Medicine, Health, Medicine D, Board on Population H, Public Health P et al. Public Health Consequences of E-Cigarettes. In: Eaton DL, Kwan LY, Stratton K, editors. Public Health Consequences of E-Cigarettes. Washington (DC): National Academies Press (US) Copyright 2018 by the National Academy of Sciences. All rights reserved. 2018.

[16] Dai X, Gil GF, Reitsma MB, Ahmad NS, Anderson JA, Bisignano C, et al. Health effects associated with smoking: a burden of proof study. Nat Med. 2022;28(10):2045–55. 36216941 10.1038/s41591-022-01978-x PMC9556318

[17] Collaborators GMD. Global, regional, and national burden of 12 mental disorders in 204 countries and territories, 1990–2019: a systematic analysis for the global burden of disease study 2019. Lancet Psychiatr. 2022;9(2):137–50. 10.1016/S2215-0366(21)00395-3 PMC8776563 35026139

[18] NIDA. Do people with mental illness and substance use disorders use tobacco more often? United States: National Institute on Drug Abuse; 2023. Available from: https://nida.nih.gov/publications/research-reports/tobacco-nicotine-e-cigarettes/do-people-mental-illness-substance-use-disorders-use-tobacco-more-often.

[19] Fluharty M, Taylor AE, Grabski M, Munafò MR. The association of cigarette smoking with depression and anxiety: a systematic review. Nicotine Tob Res. 2017;19(1,1):3–13. 27199385 10.1093/ntr/ntw140 PMC5157710

[20] Hahad O, Beutel M, Gilan DA, Michal M, Schulz A, Pfeiffer N, et al. The association of smoking and smoking cessation with prevalent and incident symptoms of depression, anxiety, and sleep disturbance in the general population. J Affect Disord. 2022;313:100–9. 35777492 10.1016/j.jad.2022.06.083

[21] Brose LS, Brown J, Robson D, McNeill A. Mental health, smoking, harm reduction and quit attempts - a population survey in England. BMC Public Health. 2020;20(1):1237. 32795286 10.1186/s12889-020-09308-x PMC7427923

[22] Taylor GM, Lindson N, Farley A, Leinberger-Jabari A, Sawyer K, et al. Te Water Naudé R, Smoking cessation for improving mental health. Cochrane Database Syst Rev. 2021;3(3):Cd013522. 33687070 10.1002/14651858.CD013522.pub2 PMC8121093

[23] (HINTS) HINTS. Health Information National Trends Survey (HINTS). National Cancer Institute; 2024. Cited 2024.

[24] Hesse BW, Moser RP, Rutten LJ, Kreps GL. The health information National trends survey: research from the baseline. J Health Commun. 2006;11(Suppl 1):vii–xvi. 16641070 10.1080/10810730600692553

[25] Hayes AF. Introduction to Mediation, Moderation, and conditional process analysis. Second ed. New York: Guilford Press; 2018.

[26] Mirbolouk M, Charkhchi P, Kianoush S, Uddin SMI, Orimoloye OA, Jaber R, et al. Prevalence and distribution of e-cigarette use among U.S. adults: behavioral risk factor surveillance system, 2016. Ann Intern Med. 2018;169(7):429–38. 30167658 10.7326/M17-3440 PMC10534294

[27] Boakye E, Erhabor J, Obisesan O, Tasdighi E, Mirbolouk M, Osuji N, et al. Comprehensive review of the national surveys that assess E-cigarette use domains among youth and adults in the United States. The Lancet Regional Health. 2023;23:100528. 10.1016/j.lana.2023.100528 PMC10366460 37497394

[28] Dutra LM, Glantz SA. High international electronic cigarette use among never smoker adolescents. J Adolesc Health. 2014;55(5):595–7. 25344030 10.1016/j.jadohealth.2014.08.010 PMC6219855

[29] Brierley ME, Gaidoni S, Jongenelis MI. Psychological distress and e-cigarette use among young Australians: an exploratory, qualitative study. Tob Induc Dis. 2024. 10.18332/tid/189395. 38899118 10.18332/tid/189395 PMC11186307

[30] Alqahtani MM, Pavela G, Lein DH Jr., Vilcassim R, Hendricks PS. The influence of mental health and respiratory symptoms on the association between chronic lung disease and E-cigarette use in adults in the United States. Respir Care. 2022. 10.4187/respcare.09579. 35440495 10.4187/respcare.09579

[31] Kassel JD, Stroud LR, Paronis CA. Smoking, stress, and negative affect: correlation, causation, and context across stages of smoking. Psychol Bull. 2003;129(2):270–304. 12696841 10.1037/0033-2909.129.2.270

